# Inflammatory-linked changes in CpG island methylation of three opioid peptide genes in a rat model for pain

**DOI:** 10.1371/journal.pone.0191698

**Published:** 2018-01-19

**Authors:** Charlotte Louise Justine Jacobi, Christoph Stein

**Affiliations:** Klinik für Anästhesiologie und operative Intensivmedizin, Campus Benjamin Franklin, Charité-Universitätsmedizin Berlin, Berlin, Germany; Universitatsklinikum Wurzburg, GERMANY

## Abstract

Expression of the opioid peptide genes *proopiomelanocortin* (*Pomc*), *proenkephalin* (*Penk*), and *prodynorphin* (*Pdyn*), in immune cells plays a key role in endogenous pain control. In a rat model of painful unilateral paw inflammation, we isolated cells from popliteal lymph nodes and evaluated the role of C_p_G island C5-methylation on the transcriptional activation of those genes. Using methylated DNA immunoprecipitation, we sorted gDNA into methylated (me) and non-me fractions and then determined the C_p_G island methylation status of each fraction *via* quantitative Real Time-PCR (qRT-PCR). *In silico* analysis by MethPrimer software identified one C_p_G island in *Pdyn* and three each in *Pomc* and *Penk*. No substantial changes in C5-methylation of any gene were observed. In conclusion, the C_p_G island methylation status does not seem to be a key regulator of opioid gene activation in immune cells during peripheral tissue inflammation.

## Introduction

The expression of endogenous opioid peptides in peripheral tissue can play an important role in pain control [1–3]. In acute inflammation, immune cells express the three opioid peptides beta-endorphin (END), Met-enkephalin (MENK) and Dynorphin A (DynA) [4–10]. Upon their release, antinociception is triggered through binding of the opioid peptides to their corresponding receptors on peripheral sensory nerve endings [11,12].

The opioid peptides END, MENK and DynA are encoded by *proopiomelanocortin* (*Pomc*), *proenkephalin* (*Penk*) and *prodynorphin* (*Pdyn*), respectively. The main expression of *Pomc* occurs in the pituitary gland where it is regulated e.g. by the transcription factors TPIT and PITX1 [13–15]. *Pdyn* and *Penk* are also predominantly expressed in the brain. In addition, all endogenous opioids are synthesized site-specifically in various organs [16–19].

So far, research has been focusing on transcriptional regulation of opioid peptide genes in the brain or on the liberation of opioids from immune cells, but little is known about the transcriptional regulation of opioid gene expression in immune cells.

All three opioid genes are, under certain circumstances, transcriptionally regulated by methylation [20–25]. The most common form of DNA methylation is the addition of a methyl group to the C5 position of cytosine within a C_p_G dinucleotide which mostly occurs outside C_p_G islands [26]. The latter is defined as a short interspersed DNA stretch containing increased amounts of C_p_G dinucleotides with a GC content > 0.5 and an observed vs. expected C_p_G ratio > 0.6 [27].

Earlier studies showed an upregulation of opioid gene expression in popliteal lymph nodes (LNs) after peripheral inflammation [10,28,29]. To investigate a possible transcriptional regulation via methylation, this paper focusses on total C_p_G island methylation of gDNA of *Pomc*, *Penk* and *Pdyn* in isolated CD45RA^±^ cells of naïve and inflamed popliteal LNs.

## Materials and methods

### Animal housing and induction of inflammation

Animal housing and experiments were carried out after approval by the local animal care committee (Landesamt für Gesundheit und Soziales, Berlin; H0370/09) and strictly met the guidelines of the International Association for the Study of Pain [30]. Male WISTAR rats (Janvier Labs) were kept in cages lined with ground corncob bedding with standard laboratory rodent chow and tap water available *ad libitum*. A light cycle of 12/12 hours light/dark, room temperature of 22°C and humidity between 40–60% were maintained. Under brief anesthesia with Forene^®^ (Abbvie) rats received an intraplantar injection of 0.15 ml Freund’s Complete Adjuvant, Modified, *Mycobacterium butyricum* (CFA) (Merck Millipore) into the right hind paw. After 96 h of inflammation, animals were killed by an overdose of inhalation anesthesia (Forene^®^). Naïve animals were left untreated.

### Preparation of CD45RA^+-^ cells from LNs

In case of CFA-injected animals, the popliteal LN of the right hind paw, and in case of naïve animals, both popliteal LNs and the cervical LNs were dissected and kept on ice in RPMI 1640 medium (Gibco). Cell suspensions were prepared by homogenizing LNs with scalpel blades and plastic pestles. Remaining cell clumps were excluded using 70 and 40 μm cell strainers. Cells were counted using a Neubauer Chamber, centrifuged and resuspended in 80 μl cold RPMI 1640 medium per 10^7^ cells. Magnetic cell sorting was performed using rat CD45RA MicroBeads, MS or LS columns, the VarioMACS Separator and the MACSmix Tube Rotator (all Miltenyi Biotec) following the manufacturer’s protocol and using RPMI 1640 medium instead of buffer. Collected cells from flow-through and eluate fractions were pelleted at 450 x g for 10 min (4°C), washed with PBS (Gibco) and stored in 1.5 ml tubes at– 80°C until further use.

### gDNA isolation and shearing

The isolation of gDNA from CD45RA^+^ or CD45RA^-^ cells was performed by adding 700 μl lysis buffer (50 mM Tris-HCl; pH 7.5); 100 mM EDTA; 100 mM NaCl; 1% SDS) and 0.5 μg/μl proteinase K to a frozen cell pellet, followed by incubating at 55°C until the pellet was lysed. The lysate was ultrasonicated for 2 x 20 s at 70% power using the SONOPULS Ultrasonic homogenizers (BANDELIN) before centrifuging for 15 min at 2500 x g. The supernatant was transferred to a fresh 2 ml tube and NaCl was added to a final concentration of 1 M. For gDNA precipitation 1 vol of isopropanol was added, mixed by inverting the tube and centrifuged for 15 min at 2500 x g. The supernatant was discarded and the pelleted gDNA was washed twice with 70% EtOH (15 min; 4°C; 2500 x g). The gDNA pellet was dried at room temperature and dissolved in TE buffer (10 mM Tris; 1 mM EDTA; pH 8).

### Methylated DNA immunoprecipitation

For gDNA shearing the gDNA was diluted in TE buffer to a final concentration of 0.1 μg/μl and 300 μl were ultrasonicated to a mean size of about 500 bp. The size of DNA was verified on 1% agarose gels using electrophoresis. Methylated DNA immunoprecipitation (MeDIP) was performed using the MethylCap kit and the Diagenode Magnetic Rack (both Diagenode) according to the manufacturer’s manual. For elution of the methylated (me) gDNA fraction only High Elution Buffer was used. To purify DNA after MeDIP the QIAquick PCR Purification Kit (Qiagen) was utilized following the manufacturer’s instructions. To elute DNA the columns were loaded twice with 50 μl PCR grade H_2_O. All gDNA samples were stored at 4°C.

### C_p_G island prediction

To predict C_p_G island positions for *Pomc*, *Penk* and *Pdyn* the MethPrimer software [31] was used. The following Ensembl sequences [32] were analyzed: ENSRNOT00000016976 (*Pomc*); ENSRNOT00000089318 (*Penk*); ENSRNOT00000037576 (*Pdyn*).

### qRT-PCR

For qRT-PCR the Mastercycler® ep *realplex*, twin.tec 96 real-time-PCR Plates, Masterclear® real-time-PCR Films (all Eppendorf) and the universal EXPRESS SYBR® GreenER™ qPCR Supermix (Life Technologies) were used with the following PCR program: 2 min 50°C; 2 min 95°C; 15 s 95°C; 10 s 61°C; 20 s 78°C; 50 cycles. An additional melting-curve-analysis was performed. A standard 20 μl reaction contained 1 x SYBR Mix, 0.2 μM primer and 2 μl gDNA/PCR grade H_2_O. Primers (TIB MOLBIOL) and sequences (forward (F)/ reverse (R)) are listed in Table 1. Rat *histone H2B type 1-A* (*TSH2B*) and *glyceraldehyde-3-phosphate dehydrogenase* (*Gapdh*) primers (Diagenode) served as positive control for me and non-me gDNA, respectively. Relative transcript levels were sustained by subtracting the threshold cycle (*Ct*) value of the total input from the corresponding *Ct* value of the gDNA-IP. Differences in gDNA levels were calculated according to the expression 2^-Δ*Ct*^.

**Table 1:** C_p_G islands in rat opioid genes and corresponding primers/ sequences.

gene	C_p_G island	genomic region	primer name	oligonucleotide sequence
*Pomc*	C_p_G1	promoter	*Pomc* C_p_G1F *Pomc* C_p_G1R	5’-GCACTTTCCAGGCACATCT-3’ 5’-CTTCTGCAACGCAACAAGC-3’
	C_p_G2	exon 1 and intron 1	*Pomc* C_p_G2F *Pomc* C_p_G2R	5’-GCGACAGGTAAGGGTGTCTC-3’ 5’-CAGAACGCCAGTCTGCATTA-3’
	C_p_G3	mostly exon 3	*Pomc* C_p_G3.1F *Pomc* C_p_G3.1R *Pomc* C_p_G3.2F *Pomc* C_p_G3.2R	5’-GTTTCCAGGCAACGGAGAT-3’ 5’-AGCACTGCTGCTGTTTCTGG-3’ 5’-GAGTTCAAGAGGGAGCTGGA-3’ 5’-GGAAGTGCTCCACCCGATAG-3’
*Penk*	C_p_G1	promoter	*Penk* C_p_G1.1F *Penk* C_p_G1.1R *Penk* C_p_G1.2F *Penk* C_p_G1.2R	5’-CCTTCGGTTTGGGGCTAAT-3’ 5’-AAAGAAGGCAAGTGTGAGGTG-3’ 5’-ACTTTCTCGGGGTTCCTCAT-3’ 5’-GCTTAGGGCAAGATTCTGGA-3’
	C_p_G2	end of exon 1 and intron 1	*Penk* C_p_G2F *Penk* C_p_G2R	5’-AGCCAGGACTGCGCTAAAT-3’ 5’-TTGGCAGAACAGTCCCTCAT-3’
	C_p_G3	exon 2	*Penk* C_p_G3F *Penk* C_p_G3R	5’-CTATGGGGGCTTCATGAGAA-3’ 5’-TCCGAGGGTAGAGACTCAGC-3’
*Pdyn*	C_p_G1	exon 4	*Pdyn* C_p_G1F *Pdyn* C_p_G1R	5’-AAACGCTATGGGGGCTTC-3 5’-ACCGAGTCACCACCTTGAAC-3’

### Statistics

The Shapiro-Wilk normality test followed by Friedman Test and Dunn’s multiple comparison test were performed to evaluate statistical significance within a group of naïve or CFA samples. To compare between naïve and CFA samples the Mann-Whitney U test was performed. *p* values <0.05 were considered significant. Data were expressed as means ± SD. Statistics were performed using GraphPad Prism version 5.02 for Windows (San Diego, California, USA).

## Results

### C_p_G island detection in rat opioid genes

**Fig 1:**
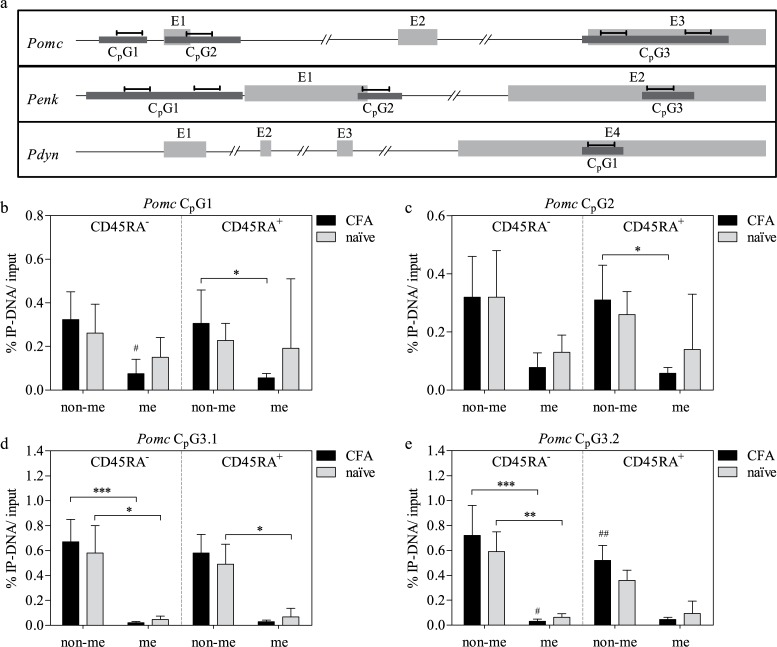
C_p_G island determination in rat opioid genes and methylation status of *Pomc* C_p_G islands in CD45RA^±^ LN cells of naïve and CFA-treated rats. Schematic structure (a) of *Pomc*, *Penk* and *Pdyn* including exons (E1-3, highlighted in grey), calculated C_p_G islands (C_p_G1-3, highlighted in black) and qRT-PCR amplicon positions (├┤). For *Pomc* C_p_G1 (b) and *Pomc* C_p_G2 (c) one amplification region was selected each, and two were selected for *Pomc* C_p_G3 (3.1 and 3.2) (d+e) using qRT-PCR. Means ± SD; *^/#^ P < 0.05; **^/##^ P < 0.01; *** P < 0.001; Friedman test with Dunn’s multiple comparison post hoc test (* non-me vs. me) and MWU test (^#^ CFA vs. naïve); n = 7 (a, b, c+d naïve), n = 8 (c+d CFA).

Using the MethPrimer software for identifying C_p_G islands within the three opioid peptide coding rat genes, three C_p_G islands were found in each *Pomc* and *Penk*, and one in *Pdyn* (Fig 1A) (Table 1).

### Methylation of *Pomc*

Under all conditions the majority of DNA fragments was found in the non-me fractions (albeit not always statistically significant) and CFA treatment resulted in decreased amounts of me DNA (Fig 1B–1E). The latter differences were statistically significant in CD45RA^-^ cells (C_p_G1+3) (Fig 1B, 1D and 1E). CFA treatment significantly increased the number of amplicons in the non-me fraction of C_p_G3.2 in CD45RA^+^ cells (Fig 1E). Differences between CD45RA^-^ and CD45RA^+^ cells were not observed.

### Methylation of *Penk*

**Fig 2:**
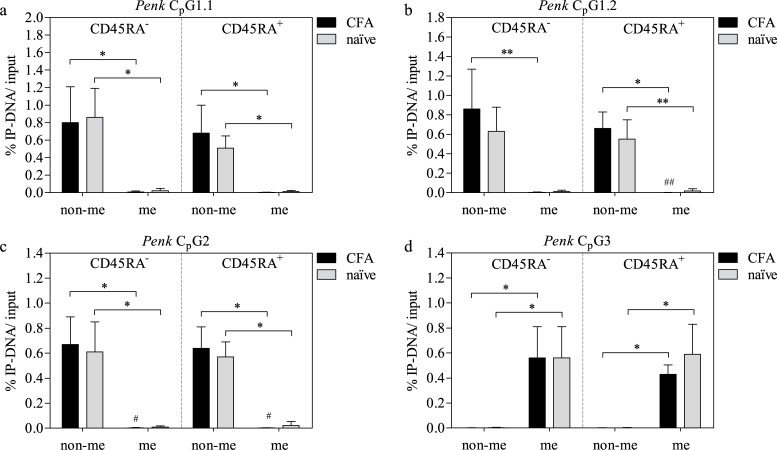
Methylation status of *Penk* C_p_G islands in CD45RA^±^ LN cells of naïve and CFA-treated rats. For *Penk* C_p_G1 two amplification regions 1.1 (a) and 1.2 (b) were selected, and one region each for *Penk* C_p_G2 (c) and *Penk* C_p_G3 (d) were chosen for qRT-PCR. Means ± SD; *^/#^ P < 0.05; **^/##^ P < 0.01; Friedman test with Dunn’s multiple comparison post hoc test (* non-me vs. me) and MWU test (^#^ CFA vs. naïve); n = 7 (a, c, d, b naïve) and n = 8 (b CFA).

The majority of DNA for *Penk* in C_p_G1+2 was not methylated (Fig 2A–2C). CFA treatment resulted in small (but statistically significant) decreases of me DNA in C_p_G1+2 of CD45RA^+^ cells and in C_p_G2 of CD45RA^-^ cells (Fig 2B and 2C). At C_p_G3 mainly me DNA was found (Fig 2D) with no differential expression resulting from CFA treatment. No differences between CD45RA^-^ and CD45RA^+^ cells were observed.

### Methylation of *Pdyn*

**Fig 3:**
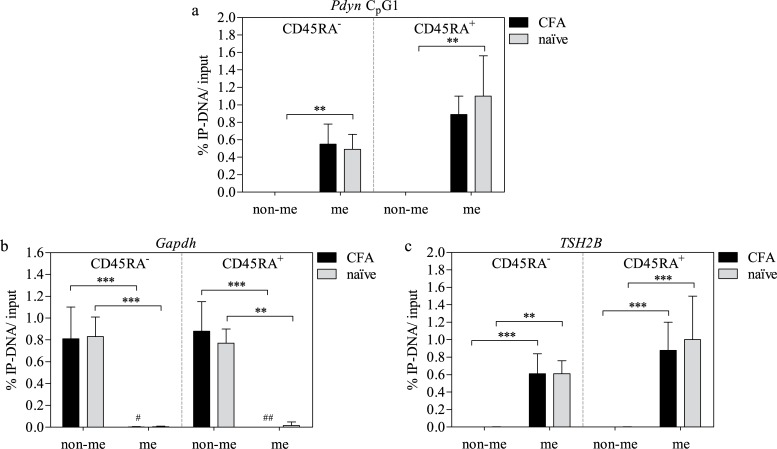
Methylation status of *Pdyn* C_p_G island in CD45RA^±^ LN cells of naïve and CFA-treated rats and MeDIP controls. Relative DNA amounts of the *Pdyn* C_p_G island DNA in each gDNA fraction were measured via qRT-PCR (a). gDNA separation via MeDIP was verified with control primers for *Gapdh* (non-me) (b) and *rTSH2B* (me) (c). Means ± SD; ^#^ P < 0.05; **^/##^ P < 0.01; *** P < 0.001; Friedman test with Dunn’s multiple comparison post hoc test (* non-me vs. me) and MWU test (^#^ CFA vs. naïve); n = 7 (a); n = 19 (b CFA); n = 18 (c CFA); n = 14 (b+c naïve).

DNA in the *Pdyn* C_p_G island almost exclusively amplified in the me DNA fraction under all conditions (Fig 3A). In naïve cells, this difference was statistically significant. No differences in methylation status between CFA-treated and naïve LN or between CD45RA^-^ and CD45RA^+^ cells were found.

### MeDIP controls

The control amplification of the C_p_G site in *Gapdh* (Fig 3B) resulted in a nearly exclusive enrichment in the non-me DNA fraction, whereas for *TSH2B* (Fig 3C) the accumulation occurred only in the me DNA fraction. No differences in methylation status between CFA-treated and naïve LN or between CD45RA^-^ and CD45RA^+^ cells were found.

## Discussion

Painful paw inflammation has been shown to increase the expression of *Pomc* mRNA and its protein product END in immune cells of LNs draining an inflamed hind paw. In 2007, Sitte et al. [10] showed the increased END production in LN cells after paw inflammation and the expression of several splice variants of *Pomc* mRNA. Maddila et al., 2016 [33] showed that mRNA expression of *Pomc* was upregulated in LN B-cells after inducing paw inflammation and that mRNA and protein expression of the POMC processing enzymes PC1 and PC2 also increased shortly thereafter. After 24 h of inflammation, a significant increase of END in LN B-cells was confirmed. To further elucidate the underlying mechanisms, the present study focused on DNA methylation, a common means of transcriptional regulation. Since not only END but also ENK and DynA were shown to contribute to endogenous pain control through immune cells [4,8] all three opioid peptide genes were investigated here.

To analyze DNA methylation not only the promoter regions were examined but also exons and introns because alterations in methylation pattern of 5´-upstream regions can also influence transcription [34–36]. This has already been shown for *POMC* by Kuehnen et al., 2012 [37], who found a hypermethylation at the boundary of intron 2 and exon 3 associated with obesity. Also *PENK* expression was shown to be regulated by methylation in the first exon *in vitro* [38].

Several forms of methylation are known, including 5-methylcytosine, 5-hydroxymethylcytosine or the rather unfrequent N6-methyladenosine. Since 5-methylcytosine is the most prevalent and the regulation of all three opioid genes has been associated with C5-(de)methylation, it was chosen for this study [23,25,39]. The *in silico* identification of C_p_G islands in all opioid peptide genes (Fig 1A) also suggests epigenetic regulation.

Methylation of C_p_G islands is generally associated with gene silencing whereas demethylation usually correlates with gene activation [40,41]. We therefore expected DNA hypomethylation in LN cells following CFA treatment. Our results show that there are no drastic differences in methylation of opioid peptide genes with or without paw inflammation, or between CD45RA^+^ and CD45RA^-^ LN cells in both conditions. Several C_p_G sites in *Pomc* (Fig 1B–1D) and *Penk* (Fig 2B and 2C) show a small but statistically significant decrease in the amount of me DNA comparing CFA-treated and naïve LN cells. Taking into account that in all cases the majority of amplified DNA was found in the non-me DNA fraction, those differences do not seem to be of biological relevance. This suggests that epigenetic regulation *via* methylation is not particularly prominent. However, a striking difference in the methylation status comparing the different C_p_G islands of *Penk* has to be noted. Finding the upstream C_p_G islands generally unmethylated but the downstream C_p_G3 highly methylated gives a hint that DNA methylation does have a certain impact on *Penk* expression.

The following limitations need to be considered: We examined the general C_p_G island C5-methylation status of the three major opioid peptide genes using MeDIP. The MeDIP assay investigates defined DNA regions for cumulations of C5-methylation, but it is impossible to look at single C_p_G dinucleotides or other methyl-associated DNA-modifications such as e.g. 5-hydroxymethylcytosine (5-hmC). Thus, only those transcriptional regulation processes can be identified, which are mediated by general C5-(de)methylation of an entire DNA segment including several C_p_G sites. However, transcriptional regulation can also be initiated by few or single C_p_G sites [42,43]. To obtain information about single C_p_G methylation, two common tools, DNA bisulfite modification or methyl-sensitive restriction enzymes could be used for future analysis, keeping in mind that both strategies also have limitations. For example, not every C_p_G site binds restriction enzymes. Bisulfite treatment would result in single C_p_G resolution. However, DNA degradation, high GC-contents and 5-hydroxymethylation hinder analysis [44]. The MethylCap kit used here is limited by the quality of DNA and the MethylCap protein. However, both are rather unlikely to influence our results. The DNA preparation was checked using agarose gels and, next to the positive controls (*Gapdh* and *TSH2B*), both me and non-me DNA sections were identified in the opioid peptide genes, thus supporting the functionality of the assay and of the MethylCap protein in particular. A false contribution of hemimethylated DNA to the results is also unlikely, since the MethylCap protein has low affinity for hemimethylated DNA [45] and mainly non-me regions were identified. PCR experiments were conducted using specific primers, designed to each span the region with the greatest C_p_G appearance. For C_p_G-islands longer than 300 bp, two amplification regions were chosen, in both cases showing the same trend in the C5-methylation status.

## Conclusion

In conclusion, C_p_G island C5-methylation is apparently not the key regulator of transcriptional activation of opioid peptide genes in immune cells from rats with acute paw inflammation. The MeDIP assay is an appropriate tool to analyze the general methylation status of specified genomic regions, as demonstrated by the opposing results for different C_p_G islands. Nevertheless, our results cannot provide a general prediction without further experiments. Investigations of single C5_p_G methylation, other methylation-based DNA modifications and CpG island shores in different stages of inflammation are necessary to fully elucidate DNA methylation as a regulating element of opioid peptide expression in immune cells.

## Supporting information

S1 Table Data sets for Figs 1–3. The table lists the individual values for each bar in Figs 1–3 including the values for means ± SD.
(PDF)
