# Supplementary material for: Inflammatory-linked changes in CpG island methylation of three opioid peptide genes in a rat model for pain
Source: PLoS One. 2018 Jan 19;13(1):e0191698. doi: 10.1371/journal.pone.0191698 (PMC5774833; doi:10.1371/journal.pone.0191698)
Supplement: S1 Table — The table lists the individual values for each bar in Figs 1–3 including the values for means ± SD. (PDF) [file pone.0191698.s001.pdf]

	Pomc															
	CpG1				CpG2				CpG3.1				CpG3.2			
	CD45RA-		CD45RA+		CD45RA-		CD45RA+		CD45RA-		CD45RA+		CD45RA-		CD45RA+	
	non-me	me	non-me	me	non-me	me	non-me	me	non-me	me	non-me	me	non-me	me	non-me	me
CFA	0,394	0,060	0,450	0,073	0,396	0,080	0,334	0,076	0,711	0,021	0,661	0,031	1,067	0,031	0,574	0,048
	0,499	0,092	0,539	0,047	0,481	0,104	0,503	0,040	0,699	0,041	0,872	0,055	0,851	0,071	0,719	0,086
	0,303	0,050	0,211	0,068	0,278	0,062	0,206	0,079	0,689	0,014	0,471	0,019	0,545	0,026	0,408	0,029
	0,356	0,023	0,286	0,076	0,403	0,030	0,323	0,073	0,762	0,015	0,512	0,029	0,716	0,024	0,657	0,055
	0,204	0,049	0,219	0,069	0,161	0,055	0,286	0,065	0,675	0,032	0,570	0,037	0,734	0,040	0,491	0,043
	0,121	0,217	0,346	0,034	0,116	0,176	0,408	0,042	0,432	0,018	0,371	0,011	0,481	0,030	0,496	0,034
	0,390	0,032	0,089	0,028	0,410	0,039	0,129	0,029	0,995	0,007	0,634	0,022	0,985	0,012	0,416	0,028
									0,436	0,015	0,552	0,028	0,393	0,016	0,373	0,038
mean	0,324	0,075	0,306	0,056	0,321	0,078	0,313	0,058	0,675	0,020	0,580	0,029	0,722	0,031	0,517	0,045
SD	0,127	0,067	0,153	0,020	0,140	0,050	0,120	0,020	0,180	0,011	0,150	0,013	0,240	0,018	0,120	0,019
naïve	0,384	0,096	0,348	0,056	0,442	0,079	0,411	0,058	0,701	0,066	0,689	0,052	0,687	0,082	0,486	0,076
	0,239	0,050	0,292	0,069	0,286	0,055	0,325	0,076	0,704	0,014	0,716	0,033	0,594	0,018	0,459	0,042
	0,231	0,129	0,148	0,913	0,299	0,125	0,186	0,576	0,476	0,058	0,282	0,218	0,604	0,076	0,320	0,313
	0,073	0,266	0,173	0,079	0,093	0,217	0,211	0,074	0,217	0,037	0,414	0,025	0,342	0,054	0,303	0,032
	0,124	0,092	0,155	0,061	0,177	0,115	0,212	0,056	0,426	0,029	0,385	0,026	0,423	0,042	0,279	0,043
	0,436	0,134	0,277	0,068	0,584	0,111	0,258	0,062	0,872	0,035	0,510	0,042	0,825	0,053	0,341	0,055
	0,338	0,286	0,199	0,097	0,340	0,197	0,235	0,083	0,692	0,093	0,445	0,073	0,684	0,110	0,314	0,091
mean	0,261	0,150	0,227	0,192	0,317	0,128	0,263	0,141	0,584	0,047	0,492	0,067	0,594	0,062	0,357	0,093
SD	0,134	0,090	0,078	0,318	0,160	0,059	0,079	0,190	0,220	0,027	0,160	0,069	0,160	0,030	0,081	0,099

	Penk															
	CpG1.1				CpG1.2				CpG2				CpG3			
	CD45RA-		CD45RA+		CD45RA-		CD45RA+		CD45RA-		CD45RA+		CD45RA-		CD45RA+	
	non-me	me	non-me	me	non-me	me	non-me	me	non-me	me	non-me	me	non-me	me	non-me	me
CFA	0,554	0,001	0,770	0,004	0,988	0,002	0,741	0,003	0,749	0,002	0,687	0,004	0,003	0,602	0,002	0,548
	0,492	0,007	0,680	0,002	0,765	0,002	0,754	0,003	0,767	0,002	0,784	0,002	0,003	0,328	0,001	0,394
	1,611	0,004	0,869	0,005	0,543	0,008	0,558	0,002	0,396	0,005	0,510	0,003	0,001	0,401	0,000	0,341
	0,481	0,005	0,442	0,004	1,611	0,004	0,846	0,001	0,965	0,002	0,670	0,002	0,002	0,426	0,003	0,444
	0,588	0,012	1,217	0,001	1,264	0,001	0,650	0,002	0,498	0,003	0,711	0,005	0,001	0,381	0,002	0,487
	0,955	0,004	0,223	0,002	0,501	0,004	0,586	0,005	0,447	0,007	0,820	0,002	0,001	0,992	0,001	0,456
	0,948	0,029	0,556	0,006	0,450	0,013	0,808	0,001	0,854	0,003	0,316	0,002	0,003	0,775	0,001	0,351
					0,773	0,002	0,305	0,002								
mean	0,804	0,009	0,680	0,003	0,862	0,005	0,656	0,002	0,668	0,003	0,643	0,003	0,002	0,558	0,001	0,432
SD	0,410	0,010	0,320	0,002	0,410	0,004	0,170	0,001	0,220	0,002	0,170	0,001	0,001	0,250	0,001	0,075
naïve	0,860	0,005	0,736	0,003	0,442	0,003	0,734	0,003	0,666	0,004	0,757	0,003	0,004	0,696	0,002	0,692
	0,992	0,004	0,528	0,007	0,775	0,004	0,825	0,009	0,843	0,003	0,673	0,006	0,001	0,154	0,003	0,846
	0,872	0,012	0,348	0,045	0,689	0,008	0,243	0,069	0,602	0,007	0,408	0,092	0,003	0,875	0,002	0,945
	0,397	0,071	0,400	0,009	0,235	0,015	0,461	0,007	0,242	0,016	0,494	0,008	0,000	0,524	0,002	0,441
	0,463	0,004	0,410	0,001	0,472	0,004	0,474	0,003	0,456	0,004	0,491	0,004	0,001	0,382	0,001	0,285
	1,322	0,008	0,623	0,006	0,900	0,007	0,634	0,005	0,971	0,008	0,645	0,005	0,006	0,452	0,001	0,411
	1,112	0,051	0,503	0,019	0,882	0,043	0,448	0,026	0,517	0,030	0,506	0,029	0,005	0,803	0,005	0,522
mean	0,860	0,022	0,507	0,013	0,628	0,012	0,546	0,017	0,614	0,010	0,568	0,021	0,003	0,555	0,002	0,592
SD	0,330	0,027	0,140	0,015	0,250	0,014	0,200	0,024	0,240	0,010	0,120	0,033	0,002	0,250	0,001	0,240

	Pdyn CpG1 CD45RA- non-me	me	CD45RA+ non-me	me
CFA	0,001	0,786	0,001	1,016
	0,001	0,394	0,001	0,982
	0,000	0,353	0,001	0,568
	0,002	0,531	0,001	0,744
	0,000	0,247	0,000	1,139
	0,001	0,831	0,001	1,052
	0,001	0,684	0,000	0,731
mean	0,001	0,547	0,001	0,890
SD	0,001	0,230	0,000	0,210
naïve	0,000	0,814	0,002	1,524
	0,001	0,376	0,001	1,078
	0,001	0,410	0,000	1,896
	0,001	0,424	0,001	0,891
	0,001	0,362	0,000	0,566
	0,003	0,408	0,001	0,762
	0,002	0,611	0,005	1,078
mean	0,001	0,486	0,001	1,114
SD	0,001	0,170	0,002	0,460

	Gapdh			
	CD45RA-		CD45RA+	
	non-me	me	non-me	me
CFA	0,773	0,000	1,238	0,004
	0,888	0,001	0,988	0,002
	0,925	0,001	0,746	0,001
	0,792	0,000	1,139	0,001
	0,828	0,002	1,034	0,001
	0,641	0,000	1,255	0,002
	0,711	0,001	1,097	0,002
	0,754	0,002	1,383	0,001
	1,135	0,003	0,828	0,001
	0,049	0,011	0,863	0,001
	1,503	0,000	0,709	0,001
	1,221	0,002	0,759	0,002
	0,706	0,001	0,792	0,001
	0,734	0,001	0,641	0,003
	0,556	0,003	0,922	0,001
	0,797	0,005	0,857	0,001
	0,806	0,003	0,400	0,001
	0,739	0,003	0,429	0,001
	0,891	0,013	0,706	0,002
mean	0,813	0,003	0,883	0,002
SD	0,290	0,004	0,270	0,001
naïve	1,078	0,002	0,857	0,001
	0,955	0,003	0,882	0,001
	0,958	0,001	0,958	0,002
	0,978	0,002	0,843	0,002
	0,716	0,003	0,800	0,092
	0,654	0,006	0,643	0,091
	0,657	0,005	0,811	0,005
	0,489	0,006	0,648	0,005
	0,677	0,001	0,636	0,001
	0,709	0,001	0,558	0,001
	1,034	0,004	0,982	0,005
	1,009	0,005	0,711	0,004
	0,913	0,015	0,781	0,014
	0,820	0,014	0,636	0,016
mean	0,832	0,005	0,768	0,017
SD	0,180	0,005	0,130	0,032

	TSH2B			
	CD45RA-		CD45RA+	
	non-me	me	non-me	me
CFA	0,001	0,903	0,001	1,503
	0,001	1,089	0,000	1,167
	0,001	0,948	0,000	0,903
	0,002	0,494	0,002	0,794
	0,002	0,533	0,003	0,803
	0,003	0,598	0,001	0,878
	0,003	0,530	0,002	0,641
	0,001	0,484	0,001	0,418
	0,000	0,471	0,001	0,734
	0,001	0,654	0,004	0,731
	0,003	0,430	0,002	0,982
	0,002	0,353	0,002	0,663
	0,001	0,322	0,001	0,999
	0,001	0,291	0,001	0,872
	0,000	0,675	0,002	1,721
	0,000	0,851	0,002	0,808
	0,001	0,576	0,000	0,602
	0,002	0,773	0,000	0,550
mean	0,001	0,610	0,001	0,876
SD	0,001	0,230	0,001	0,320
naïve	0,002	0,828	0,001	0,971
	0,001	0,811	0,002	1,034
	0,001	0,450	0,001	1,291
	0,001	0,444	0,002	0,775
	0,003	0,834	0,002	2,367
	0,002	0,663	0,002	1,662
	0,001	0,684	0,001	0,831
	0,000	0,576	0,002	0,666
	0,001	0,524	0,001	0,634
	0,001	0,439	0,001	0,537
	0,004	0,458	0,001	0,909
	0,003	0,455	0,001	0,602
	0,005	0,680	0,006	1,074
	0,003	0,641	0,006	0,636
mean	0,002	0,606	0,002	0,999
SD	0,001	0,150	0,002	0,500
